# How foreign and domestic pressures on NGOs shape service provision for HIV in Tajikistan: a qualitative analysis

**DOI:** 10.1186/s12889-026-27450-w

**Published:** 2026-04-21

**Authors:** Tara McCrimmon, Nikolay Lunchenkov, Lisa R. Metsch, Jonbek Jonbekov, Farzona Sangova, Mastura Sirojiddinova, Stevan M. Weine, Morgan M. Philbin

**Affiliations:** 1 Department of Psychiatry, Yale University School of Medicine, New Haven, CT USA; 2 TUM School of Social Science and Technology, Technical University of Munich, Richard-Wagner-Straße 1, Munich, 80333 Germany; 3 Department of Sociomedical Sciences, Columbia University Mailman School of Public Health, New York, NY USA; 4 Prisma Research Center, Dushanbe, Tajikistan; 5 Avicenna Tajik State Medical University, Dushanbe, Tajikistan; 6 Center for Global Health, University of Illinois College of Medicine, Chicago, IL USA; 7 Division of Health and Society, Department of Medicine, University of California San Francisco, San Francisco, CA USA

**Keywords:** Eastern Europe & Central Asia, HIV, Service access & delivery, Civil society

## Abstract

**Background:**

Non-governmental organizations (NGOs) are key to an effective HIV response due to their ability to reach hidden or marginalized populations that may avoid interactions with state-run clinics and services. Over the past decade in the Eastern Europe and Central Asia (EECA) region, funding for HIV programming has decreased, constricting NGO operations. Concurrently, government regulation has increased, including laws that complicate how organizations receive international funding. This study explores how this changing environment impacts NGO survival and HIV-related service delivery in Tajikistan.

**Methods:**

We conducted 28 in-depth interviews in 2024 with key informants across Tajikistan in government, state-run medical clinics, and the civil society sector (NGOs and partner organizations). Interviews focused on barriers to delivering HIV prevention services, including external structural constraints that impacted organizational funding and operations. Interviews were recorded, transcribed, translated, and analyzed using thematic content analysis techniques.

**Results:**

NGOs balanced obtaining funding for delivering HIV prevention and care services with pressures from both international donors and the Tajik government. Participant accounts described three primary challenges to the work of NGOs and partners in Tajikistan’s civil society sector. These included: 1) adapting programming to address emergent needs as the HIV epidemic shifts (e.g., to new populations); 2) aligning program requests and reporting requirements from international donors with local priorities and needs; and 3) navigating an increasingly restrictive legal environment, including perceived increases in pressure coupled with a lack of support from international partners.

**Conclusions:**

Our findings highlight the structural challenges Tajikistan’s NGOs must navigate to provide HIV prevention services. There is an urgent need to strengthen the capacity and autonomy of the civil society sector, and to increase its ability to reach key populations.

**Supplementary Information:**

The online version contains supplementary material available at 10.1186/s12889-026-27450-w.

## Background

Eastern Europe and Central Asia (EECA) is one of the few global regions where HIV incidence continues to rise: There were 48% more annual incident cases in 2021 than in 2010 [1]. Transmission dynamics in EECA have shifted in the past decade; between 2010 and 2022, new HIV infections decreased by 10% among people who inject drugs (PWID), but rose in other key populations, including a 74% increase among sex workers (SW) and a 144% increase among men who have sex with men (MSM) [2]. Tajikistan accounts for a small portion of the EECA region’s HIV epidemic, with fewer than 1,000 new cases identified per year. However, it has a high HIV prevalence among key populations: 8.9% among PWID (population size estimate: 18,000), 2.9% among SW (18,400), and 4.3% among MSM (13,400) as of most recent available key population surveillance efforts in 2022 [1]. The country’s economic and political reliance on Russia, and an increasingly conservative and religious culture, make HIV prevention and treatment in Tajikistan uniquely challenging.

Non-governmental organizations (NGOs) play a vital role in the global HIV response, by both providing direct services and helping clients navigate through formal medical systems [3–5]. With their staff and leadership often drawn from affected communities, NGOs are well-equipped to address the specific needs and priorities of key populations impacted by HIV [6, 7]. Key populations with and without HIV often prefer NGOs, where disclosing stigmatized or criminalized behaviors such as substance use or sex work is less likely to result in prosecution, government surveillance, or loss of housing, employment, or parental rights, as it may at state-run clinics [4, 7–9]. At the same time, NGOs are more likely to reach and engage clients from marginalized groups at risk of HIV, which makes them valuable in achieving state-set priorities and activities for epidemic control [10, 11].

**Fig. 1:**
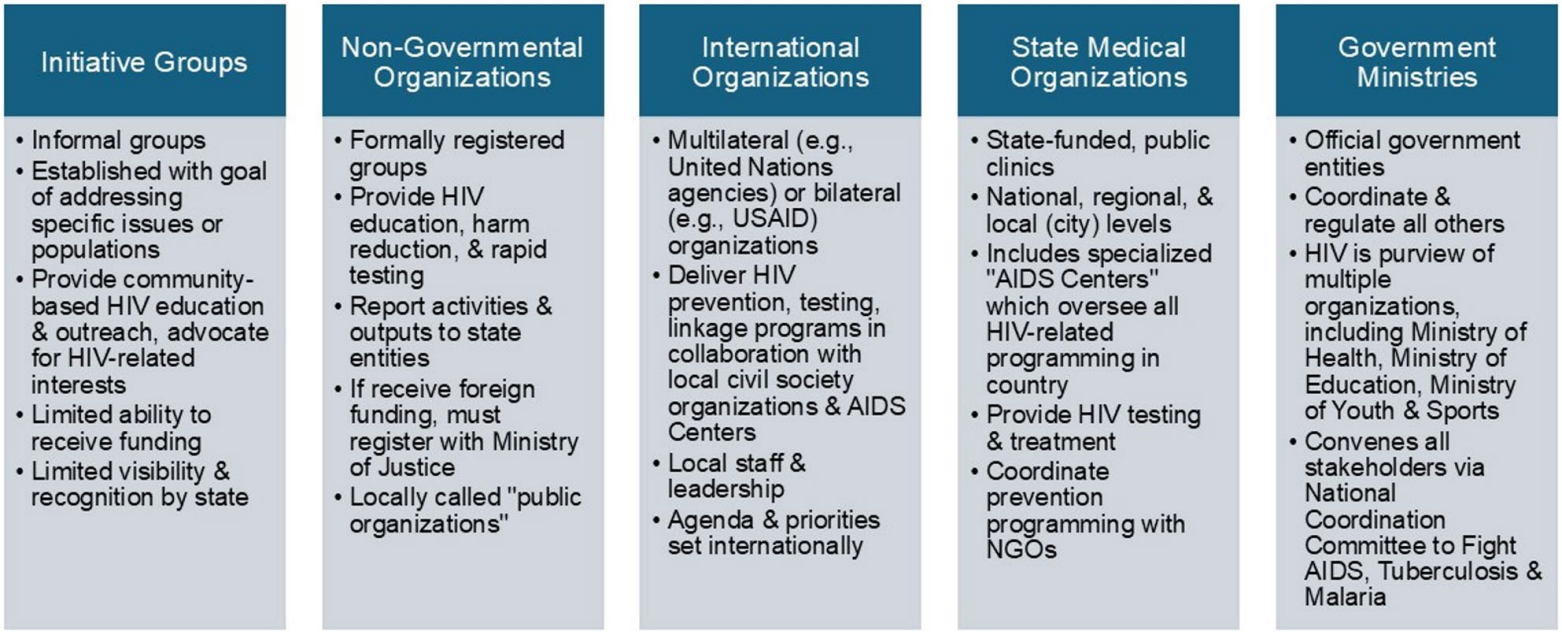
Descriptions of the entities involved in Tajikistan's HIV response

Tajikistan’s HIV response landscape includes a wide range of organizational actors as outlined in Fig. 1. HIV is included in the mandate of several government ministries, including Health, Education, Youth and Sports, and their regional and local affiliates, including local public health departments. Like other countries in the EECA region, Tajikistan inherited a centralized and siloed healthcare system from the Soviet Union, with HIV treatment provided through specialized state-funded clinics known as “AIDS Centers.” With supervision from the Ministry of Health, the National AIDS Center coordinates and collects official data on all HIV prevention and treatment activities. NGOs, which are officially called “public organizations”, do not provide HIV treatment directly but can link communities with formal medical services. NGOs can provide information and education about HIV, harm reduction services (condom distribution and syringe services) and rapid testing for HIV. Tajikistan’s Law on Public Associations sets out the laws governing registration and operation of NGOs [12]. Additionally, Tajikistan has informal “initiative groups” with more limited activities. Finally, international organizations in Tajikistan include both multilateral bodies (e.g., UN agencies) and bilateral agencies (e.g., USAID), which may have local offices and leadership, but operate under priorities and agendas determined outside the country.

Previous research on NGOs in the EECA region has explored their status in a changing political landscape. After the dissolution of the Soviet Union in 1991, Western governments promoted the development of civil society as a key step in the region’s democratization, and established NGOs as a vehicle for this process [13]. A number of bilateral and multilateral organizations established themselves in Tajikistan during and following the country’s 1992–1997 civil war; this led to a proliferation of local NGOs who could partner with international donors to provide direct services [14]. In addition to mission-specific activities, such as nutrition, education, or health, Western nations framed NGOs as an alternative to government structures and potential venues for civil society to take root [10]. However, the resulting reality has been more complex; research from Tajikistan, as well as other countries in the region such as Poland and Kyrgyzstan, shows that regional NGOs must often balance donor expectations and requirements with local pressures and needs [10, 13, 15]. While Tajik NGOs are adept at using language that describes “building civil society” to donors, in practice they often focus on technical service delivery rather than activism or advocacy for government reforms or civil rights [15]. Even so, NGOs in the EECA region still face many barriers to operations, and they are frequently marginalized in their countries’ HIV responses [4, 16].

In the past decade, the landscape of HIV prevention service delivery in Tajikistan has shifted significantly, marked by decreasing international HIV funding [1], and an increasingly restrictive operating environment for NGOs. This includes a series of amendments to Tajikistan’s Law on Public Associations, beginning with one in 2015 which required NGOs to disclose their foreign funding to the Ministry of Justice [12]. This was followed by a 2019 amendment requiring NGOs to post their financial information online [12, 17], and a 2021 amendment requiring that staff of foreign organizations register with the Ministry of Foreign Affairs [17]. This increased oversight is part of a broader regional constriction of the civil society sector, often based on claims that foreign-funded NGOs may support terrorism or anti-government activities [18–20]. In former Soviet states, these laws are frequently influenced by Russia’s 2012 “Foreign Agent” law, even if not explicitly labeled as such. These laws discourage NGO formation and activities through increased regulatory burden. Research on the aftermath of Russia’s 2012 “Foreign Agent” law identified a number of ways in which NGOs adapted their programming to continue services—for example by avoiding any activities or actions that could be seen as political, separating into multiple organizations to ensure some functions remained protected, or purposely losing formal registration status [18]. However, less research has explored the impacts of similar legislation in other EECA countries, including in Tajikistan. Over the past five years in Tajikistan there have been large-scale NGO closures across all fields, including local media reports of 700 closures in 2022 and 2023 alone [21]. By 2024, only nine HIV-related NGOs were registered with the Ministry of Justice; this number is inflated by inclusion of research organizations (like the Prisma Research Center where this study was conducted) in addition to direct service organizations.

Little research has explored how NGOs balance international and domestic expectations within Tajikistan’s national HIV response, and how recent decreases in funding and increases in state oversight may constrain their operations and ability to deliver HIV prevention services, particularly when they serve socially stigmatized and marginalized groups. The purpose of this paper is to examine international (funding streams and priority populations determined by international and multilateral organizations) and domestic structures (oversight, administrative burdens, and activity limits based on sector), and how they constrain NGOs from providing effective HIV programming, including education services, distributing condoms, conducting testing, and linking clients to care and treatment.

## Methods

As part of a larger qualitative study on barriers to HIV prevention among Tajik youth, we conducted and analyzed in-depth interviews with 28 key informants recruited from across Tajikistan. Within the scope of the larger research study, interviewees consistently described challenges faced by the civil society sector, which became the focus of this paper. The study leveraged the variety in cultural (US and Tajik) and disciplinary (medical and public health) perspectives in developing research questions of interest and guiding data interpretation. Procedures were approved by the Institutional Review Boards at the University of Illinois at Chicago, the Prisma Research Center in Dushanbe, and Columbia University. Ethics review at all three sites was conducted in compliance with applicable US federal regulations and was guided by the ethical principles contained in the Belmont Report and the Helsinki Declaration.

### Conceptual framework

This paper leverages the Practical, Robust Implementation and Sustainability Model (PRISM) [22] to identify and understand the factors that impact delivery of HIV services by Tajikistan’s NGOs, particularly those related to an organization’s external context, including policies and regulations, as well as its relations with other organizations. These are assumed to impact program implementation and sustainability but are understudied, due to methodological challenges in isolating them from other determinants and operationalizing them as measurements [23]. This is particularly true for research conducted in low and middle income country (LMIC) settings, where healthcare systems and norms are very different than those in high-income countries [24], and where international organizations provide a large portion of programming for HIV. With limited evidence from the EECA region, our study contributes to understanding how various government and international actors influence HIV prevention programming in this context. As detailed in Table 1, we utilized the PRISM framework in developing our interview guide, informing questions about external context, as well as the other three key domains (implementation and sustainability infrastructure, intervention, and recipients). As described further below, we also used PRISM to develop our initial set of a priori codes during the analysis, although our iterative codebook creation process allowed us to develop inductive codes beyond this framework.

**Table 1:** Sample interview questions^A^

Topic	PRISM Domain/Construct	Sample Questions
Factors which facilitate HIV program delivery at NGOs	External/Regulatory environment	*What programs of yours do you consider to have been successful?* *What factors (policies/procedures/staffing/leadership) contributed to these successes?*
Factors which constrain HIV program delivery at NGOs	External/Regulatory environment	*What programs have been challenging to deliver?* *What factors (policies/procedures/staffing/leadership) contributed to these challenges?* *What strategies did you use to overcome these challenges?*
Ease of starting new programs at NGOs	Intervention/Readiness	*If you heard about a new training, program, or service that you think would be good for your clinic/organization, can you describe the steps you would take to realize it?* *Who would you need support/approval from? Apart from funding, would you need additional staff trainings, staff, space to implement a new program?*
How NGOs adjust international programs	Intervention/Usability and adaptability	*What are your opinions on using internationally-developed (outside of Tajikistan) HIV prevention programs in your organization? What adjustments would you need to make to these programs to make them appropriate for Tajikistan or appropriate for your organization?*
NGO staff motivation and dedication	Recipients/Staffing and incentives	*What motivates the staff at your organization to do the work that you do?*
Continuity of NGO funding and sustainability	Recipients/Expectation of sustainability	*When new programs are introduced, is there an expectation that they will be maintained in the long run (5* + *years?)*
NGO’s evaluation of program success	Implementation and sustainability infrastructure/Performance data	*What data would you need to determine whether a new program was “successful”? What does “success” of an HIV program mean to you?*

^A^The full interview guide is available as a supplementary file

### Participant recruitment and eligibility

Between February and May 2024, we conducted 28 key informant interviews. As part of a larger study on HIV prevention among youth, our sample was purposively selected to capture diverse perspectives across multiple settings (educational, medical, community). We targeted individuals who: 1) were 18 + years old; 2) had conducted work focused on HIV for at least one year; and 3) were able to complete an interview in Russian, Tajik, or English. An eight-member study advisory board (consisting of key partners from medical, government, and community organizations who provided professional guidance on the research design and conduct) helped identify the following organization types for recruiting key informants: 1) Tajik government institutions, including the Republican AIDS Center, the Ministry of Education, the Ministry of Youth and Sports, 2) City and regional AIDS Centers; 3) Other medical clinics, including family medicine and narcology centers in Dushanbe; and 4) Civil society and partners, including national and regional NGOs, informal initiative groups, and international organizations which operated in multiple countries and partnered with local ones. We used recommendations from study advisory board members and participants themselves to identify individuals who could provide detailed and rich information on HIV prevention programming in Tajikistan. The research team approached potential participants by phone or email, confirmed that they met the above criteria, provided a description of the study, and assessed their interest. Advisory board members facilitated introductions where possible to these individuals. As discussed in our limitations section, while we reached out to participants at UN agencies, none responded to our requests to be interviewed. Incorporating two concepts of data saturation and sufficiency, [25, 26] the research team stopped recruitment when existing narratives had addressed our research questions and themes began to repeat between interviews.

### Measures

As stated above, these analyses were part of a larger study on HIV prevention among Tajik youth. We developed a semi-structured interview guide, informed by the PRISM framework (see above and Table 1). We asked participants about challenges they faced to delivering HIV-related programming, and how requirements and regulations from both the Tajik government and international donor organizations contributed to these challenges. After developing the interview guide, we piloted it in a partner organization setting and made necessary adjustments. Sample questions are provided in Table 1. Interviewers were trained to probe participant responses and to use follow-up questions to collect as many details as possible. Participant sociodemographics were collected at the end of the interview.

### Data collection and analysis

Two research assistants with medical training (one second year medical student and one medical school lecturer) were trained to conduct interviews in Russian and Tajik languages; the first author attended all interviews and conducted three of them in English. Key informants selected a time and private place for their interview, most commonly their place of work. Four participants in Dushanbe who lacked a private office conducted their interviews in the Prisma Research Center office, and four participants were interviewed by phone. Participants were provided with an information sheet about the study and we obtained verbal informed consent before beginning each interview. Each interview lasted 45–60 min, and was audio recorded. Participants received 200 somoni (approximately $20) for their time.

Audio files were transcribed in the language they were conducted and translated verbatim by the research assistants, who removed the names of individuals and organizations during this process. Due to the politically sensitive nature of our findings, we have excluded individual participant identifiers such as gender, age, and location, and identify participants only by their role and organization type. The first author developed an initial codebook based on the PRISM framework and updated and refined these codes over three rounds using three English-language interview translations. Spot checks of translated quotes and key points, along with consultations with bilingual research team members, were conducted to ensure accuracy and clarity. Between each round of coding, the codebook was updated in consultation with the broader research team. The final codebook was then applied to all 28 English-language transcripts using Dedoose software. A thematic analysis approach was used to analyze the data [27], in which the first author reviewed excerpts and organized them into overarching themes, which each extended over multiple PRISM domains. Data interpretation involved iterative discussions with other members of the research team in Tajikistan and the US to cross-check initial impressions against alternative cultural and professional framings, explicitly addressing Western-centric assumptions and siloed clinical perspectives.

## Results

Our full sample included 28 key informants. This paper centers the accounts of the 17 participants representing the civil society sector, including initiative group members (*n* = 4), NGO staff (*n* = 10), international organization staff (*n* = 1), and consultants who had experience across local NGOs, international organizations, and educational institutions (*n* = 2). Demographic and professional characteristics are described in Table 2. These participants described unique organizational challenges that span multiple constructs of the PRISM framework – e.g., policy-driven constraints on their HIV program delivery (External/Regulatory environment) and impacts on adopting or adapting new HIV programming in their organization (Intervention/Readiness, Usability and adaptability), sustaining staff motivation and commitment (Recipients/Staffing and incentives), and assessments of program success (Implementation & sustainability infrastructure/Performance data). However, where specifically noted, views from the full sample of 28 interviewees are also incorporated for essential background and context.

**Table 2:** Key informant sociodemographics

	*N* (total = 28)
City	
Dushanbe	18
Khujand	4
Khorogh	3
Bokhtar	3
Average age (mean [SD])	45.96 [10.99]
Gender	
Male	14
Female	14
Ethnicity	
Tajik	24
Pamiri	2
Uzbek	1
Russian	1
Highest education completed	
9th grade	1
Technical or vocational school	1
University (bachelors)	4
University (masters or higher)	22
Has a medical degree (MD)	9
Average years worked in HIV (mean [SD])	15.68 [8.23]
Organization type	
National, regional, or city AIDS center	4
Other state-run medical center (e.g.,family medicine, narcology)	4
Government body (national or regional)	3
Civil society or partner organizations:	
Initiative group	4
Non-governmental organization	10
International organization	1
Consultant	2

Participants from all sectors described how HIV prevention work in Tajikistan was most frequently funded through collaborations with bilateral and multilateral donor organizations; these included the US Agency for International Development (USAID) and the President’s Emergency Plan for AIDS Relief (PEPFAR), UN agencies like the United Nations Children’s Fund (UNICEF) and the United Nations Development Programme (UNDP), The Global Fund, as well as smaller private foundations like the Elton John AIDS Foundation. They noted how international funding was commonly used to implement short-term projects or programs. Larger multilateral organizations (like UNICEF and UNDP) tended to collaborate directly with state institutions such as the National AIDS Center, while smaller NGOs and initiative groups were often subcontractors of these projects, or NGOs sought direct funding from lesser-known foreign funders. State medical institution staff reported that The Global Fund covered many antiretroviral therapy (ART) medications (including pre-exposure prophylaxis), HIV test systems, and harm reduction materials such as syringes and condoms.

Participants from all sectors noted significant reductions in civil society-led HIV programming over the past ten years, due to reductions in grant funding from international donors and government pressures to close individual NGOs. As one NGO representative described:

*And there were fewer grants, and organizations that worked in this [area], after they experienced this [government] pressure… and [then] closed. They just shut down. (Participant A, Director, NGO)*

Civil society sector representatives described experiencing three primary challenges in this changing environment: 1) they were disincentivized from adapting programming to address HIV epidemic shifts to new populations; 2) they struggled to align program requests and reporting requirements from international donors with priorities and needs that they observed on the ground; 3) they faced an increasingly restrictive legal environment, including increased national government regulation and a lack of support from international donors.

### Service gaps for emerging key populations

Civil society representatives described a climate in which NGOs had to choose between serving socially stigmatized groups like MSM and maintaining their own financial stability and legal standing. They believed that widespread HIV-related stigma in Tajik society, coupled with the association between HIV and criminalized and marginalized groups, discouraged the government from actively supporting organizations who served these populations:

*The [NGOs] who provide HIV services, it doesn’t mean that they work just with the general population, right? They are targeting the most at risk population[s], and some of these most at risk population[s]are not liked by our government, such as MSM, those who use drugs, and people who practice commercial sex… so all of these groups that HIV service organizations worked with, these organizations were forced to close. Otherwise, they were just punished… this is also I think why HIV cases are increasing, rising. Because less work is done now with [key] population[s]. (Participant C, Independent Consultant)*

No participants in our sample mentioned officially registered NGOs that targeted MSM or other LGBTQ + individuals. Where services for MSM did exist in Tajikistan, they were increasingly forced underground into initiative groups that operate without legal registration. However, all participants from initiative groups and several from NGOs noted that these initiative groups face major obstacles when it comes to their operations, including an inability to accept funding, sign contracts, or officially work with international donors. Some NGO staff noted that they had actively resisted expanding their services to MSM to protect their organization:

*If it's for HIV prevention, drug use, [sex workers], then we don't ask for any documents. Only we have one taboo…MSM, a closed topic for us, we do not want to work in this direction… We are not saying that we expel [MSM clients], we redirect them [to other organizations]… we talk to them calmly, we just redirect. (Participant A, Director, NGO)*

The self-imposed restrictions described by this participant went beyond refusals of service by individual staff and extended to strategic organizational decisions. This climate even shaped funding choices, as another organization described being hesitant to pursue grants associated with LGBTQ + work:

*Participant D, Director, NGO: We ourselves look at the [call for applications] and choose whether it suits us or not. For example, the [call] was submitted by [international funder], we read their requirements, policies and priorities and realized that this was not the right fit for us… This is a feminist women's organization, including women and the LGBT community. Although we are a women's organization, we are not so radical, so this does not suit us.*

*Interviewer: Are you legally allowed to work with LGBT organizations?*

*Participant D: It is permitted by law but not recommended.*

The euphemistic term "not recommended" implies government or societal pressures which operate through informal channels. This participant's account suggests organizations may forgo funding options focused on marginalized populations to avoid subjecting themselves to additional government oversight or societal criticism and potential harassment.

Even as civil society sector organizations avoided working with marginalized groups, public clinics offered limited or poor-quality services. All key informants described how high levels of HIV-related stigma in primary care and dental clinics deterred key population groups from seeking care. They also shared how, beyond medical settings, the government’s HIV response employed punitive measures, including criminalizing HIV transmission, while neglecting the structural and social needs of MSM or other key population communities. NGOs were therefore the preferred avenue for key populations to access support and resources.

### Challenges to working with international donors

Most civil society sector participants described complex relationships between Tajik NGOs and their international donors, which were often characterized by dependence on a rapidly shrinking pool of foreign funding. Participants whose organizations had received funding from international donors described challenges aligning donor priorities with on-the-ground realities. Several emphasized the uniqueness of Tajik culture, and the necessity of careful cultural adaptation prior to implementation:

*The projects that we do in Tajikistan… 90% are international… We adapt in context, communication messages, for example, facilitation of the trainings, any materials that we receive, for example, from [name of international partner organization]. During [their] development they [may] be targeted to some African countries, or South Asian countries, it doesn’t work in Tajikistan. We have another mentality... (Participant E, Project Manager, International Organization)*

Participant from all sectors described how HIV work in rural areas required heightened cultural sensitivity around topics such as sexual behaviors and condom use. One civil society sector participant described how even their NGO’s urban program staff faced challenges gaining local trust in these areas. Having had these experiences, this organization described the necessity to push back and negotiate with international donors:

*We directly told the donor that the program that you are implementing, the result that you want, it is in principle feasible, but there are certain difficulties… and if you agree to implement your program, taking into account our recommendations, then great, but if you want to carry out, limit your programs, without taking into account our recommendations, the probability of the project implementation will be very difficult in [some] places and sometimes not feasible at all. (Participant F, Director, NGO)*

However, it was not clear that all NGOs felt empowered to negotiate with their international donors. A few participants representing NGO and international organizations described unilateral adjustments to foreign programming and materials, such as curtailing in-depth discussions of HIV sexual transmission and condom use or implementing gender-segregated programming. Another NGO representative described frustrations their organization encountered with monitoring and evaluation processes implemented by donors:

*Until 2015, no one talked about having a huge [program] coverage, there was a basic requirement for quality. After 2015, donors put everything the other way around, the main thing for us [to provide] is coverage, and then quality… I will not say that there were fewer resources, the resources were the same, huge resources were spent. But the requirement was aimed at a large coverage… They did not say that quality was not needed, but they pushed us to give more… (Participant A, Director, NGO)*

Finally, most NGO representatives noted that the instability of the funding provided by international organizations could negatively impact the quality of services provided. One civil society representative said:

*Year after year, the number of donors is decreasing, the main burden [of providing services] goes to the Global Fund, which in turn also says that… the state itself should do it... The state says ‘We are doing it,’ but reports say something else. There are contradictions… Not one organization has its own office, they all [rely] on grants. Today, this grant-giver will leave, then everything [will be] closed. (Participant G, Director, NGO)*

This participant believed that government organizations were not assuming the responsibilities of the NGOs that had shuttered, resulting in an overall reduction in service provision.

### Pressures on the NGO sector through government regulation

Several civil society sector participants believed that the Tajik government’s oversight of NGOs, as stipulated in its Law on Public Associations and subsequent amendments [12], had been used for dismantling the infrastructure essential for HIV prevention work. One representative described how NGO leadership could be pressured into closing their organizations:

*So, I say, who cares, because of my social work, people came to my house and put pressure on me. Why do I need this? [We’ll] just shut [the NGO] down, and that's it. (Participant A, Director, NGO)*

This participant framed this personal intimidation of NGO leaders as a deliberate strategy intended to create a chilling effect throughout the civil society sector; by targeting not only organizations, but also individuals, it made the work of civil society personally dangerous and unsustainable. Other civil society sector participants described how, when organizations managed to continue their work, it was often through transitioning their organizations from formal, registered NGOs to informal initiative groups:

*In fact, the civil society organization in [region] is closed, that is, those who work on the prevention of drug addiction, HIV, all social diseases, are closed, but we work as an initiative group, we are working now mainly for human rights. (Participant B, Director, Initiative Group)*

As noted above, initiative groups in Tajikistan operate in legal limbo; while they may have more operational freedom and less government oversight due to their informal status, they are not eligible for many international grants and may rely on local contracts to fund activities.

Several civil society sector representatives believed the state was primarily concerned with consolidating their power and control over NGOs, rather than working with them as equal partners to deliver effective HIV programs. One participant expressed anger over this dynamic, describing how the state would occasionally employ accusations of "foreign agents" as an excuse to stop organizations that were doing valuable work:

*[The government] is used to [NGOs] doing everything for them… they still come and say “You, you are foreign agents!”. I’ll tell you what kind of foreign agent I am! While you were sleeping, we raised this issue [of HIV], thanks to the United States, to European countries… it was thanks to their help, their humanitarian assistance, their consultations and their financial assistance, that we now live here… (Participant B, Director, Initiative Group)*

This participant emphasized the hypocrisy of a system that depends on NGOs and international partnerships for HIV prevention funding and services, then condemned these partnerships as foreign interference.

Yet another participant shared a story of how, when local organizations appealed to their international partners for political backing in advocating against the dismantlement of NGOs, they were met with resistance:

*I was member of this National Coordination Committee, and two times the issue -- of local NGOs being forced to close and having problems with implementing HIV prevention projects -- was raised… but because the head of [the Committee] and the ministries were really against even listening to this issue, the discussion was stopped. I had discussion with [international partners], “We should not be [complacent], okay, you are in different position than me. I’m local. Yes, I’m head of the organization, but still, I’m local … but I’m not getting support from you, so let’s [join] together...” And unfortunately, the answer was “No, it’s too serious, it’s a government issue, we’ll still be advocating, but not really”… So nobody really wanted to take leadership… I always felt sorry [that] the international organizations [weren’t] active to support local NGOs. That’s why very few survived. (Participant C, Independent Consultant)*

This participant believed that international partners’ reluctance to act left local civil society organizations operating without resources or protection in an increasingly hostile environment, contributing to the NGO closures seen over the prior decade.

## Discussion

This study helps fill a critical gap in the understanding of how national governance and international pressures affect NGO-led HIV programs and service delivery in an EECA setting. Participants in our sample representing the civil society sector perceived an increasingly hostile atmosphere and challenging regulatory landscape in which their organizations operated, with overlapping pressures from the Tajik government and international donors. Perceived threats included less international funding and more demands from donors, as well as government accusations of being “foreign agents” and forced closures of NGOs. Participants described how several organizations had been unable to withstand these threats, and believed their closures had limited access to services for key populations and eroded longstanding institutional and individual expertise in the overall HIV response. Their accounts also describe how organizations were incentivized to reorient their work and shift from evidence-based programming to politically safe programming on other topics or with general populations. This could result populations with a growing HIV prevalence (e.g., MSM) [2] receiving unstable and under-resourced services. This creates critical gaps in HIV prevention coverage precisely where transmission is most likely to occur, which could undermine the overall effectiveness of the national epidemic response.

In ideal public health programming, epidemic shifts would be addressed through organizational expansion or the creation of new, specialized NGOs that serve emerging key populations like MSM. However, our findings show how both these pathways are difficult for NGOs in Tajikistan. Many of the NGOs in our sample focused on people who inject drugs, reflecting both the regional history of the HIV epidemic and their community-based origins and specialized expertise. In spite of the 144% increase in HIV incidence among MSM from 2010 to 2022 [2], existing NGOs described avoiding expanding services for MSM and LGBTQ + individuals due to government and societal pressures to avoid this key population. Meanwhile, participants believed that regulations and oversight presented barriers to establishing new NGOs and downgraded several existing NGOs into informal initiative groups – shifting them from professional organizations to volunteer networks with limited capacity. This perceived erosion of civil society infrastructure may weaken Tajikistan’s ability to respond to an evolving epidemic. An effective HIV response requires organizations that can innovate and adapt programming to meet immediate needs, including as they shift across populations. For this to happen, governments must foster a political environment that allows civil society to function autonomously, rather than imposing high levels of bureaucracy and regulation [28, 29].

Our findings highlight how regulatory oversight is an instrument of power, and how this shapes HIV-related services. The Tajik government’s use of "foreign agent" rhetoric as described by our participants creates a paradoxical situation in which local civil society organizations are expected to address public health challenges that the state cannot manage, yet face restrictions in forging international partnerships necessary to fund effective approaches. Consistent with prior literature [10, 11, 30], some civil society representatives in our sample noted that the Tajik government viewed NGOs as threats tied to Western democratization agendas. However, these participants rejected this characterization and emphasized their technical service delivery role. This suggests that Tajikistan’s 2015 amendment to the Law on Public Organizations has introduced burdensome registration requirements on NGOs receiving foreign funding. Unlike research from Russia which suggests similar laws had limited impact [18], civil society sector representatives in our sample described how many HIV-focused organizations closed following these restrictions. Democratic governance has been associated with success along the HIV care cascade, with one of the hypothesized mechanisms being freedom of civil society operations [31]. Tension between civil society and the state can hinder effective HIV responses, as evidenced by contrasting outcomes in Brazil (where NGOs are strongly supported) and Russia (where restrictions resemble Tajikistan’s) [32].

Participants also believed that dwindling international financial support was exacerbating NGO closures independent of government restrictions, a process which is likely to be accelerated given the 2025 dismantling of USAID and other foreign aid cuts under the second Trump administration. The US was a key foreign aid provider to Tajikistan’s HIV/AIDS programs, providing $3.4 million in 2024 [33], which accounted for approximately 20% of the country’s total HIV/AIDS budget according to UNAIDS estimates [34]. This made the US the second largest donor after the Global Fund, which covered approximately 40% of Tajikistan’s total HIV/AIDS budget, primarily funding ART medication and other supplies [34]. In March 2025, the initial impact of these cuts included the closure of two local NGOs that conduct community outreach, and the suspension of over 75 medical, technical, and community outreach staff [35]. Per UNAIDS reporting, these organizations reopened in December 2025, albeit at reduced capacity [36]. Scoping reviews have found that without planning, leadership, and preparatory investments in local systems and organizations, donor withdrawal can lead to service disruptions; examples in the HIV sphere have shown that key populations are most impacted by such cuts [37]. Civil society representatives in our sample noted that the loss of NGO expertise and their unique positioning as community liaisons, creates significant gaps in the HIV response that the government institutions are unlikely to fill themselves. NGOs are already marginalized within centralized government decision making bodies in Tajikistan, and stronger integration of NGO perspectives and priorities into state planning is needed. Reforms could cultivate a more supportive environment where NGOs are financially supported and unrestricted. However, there remains a lack of research into how to increase NGO involvement within government systems that civil society deems hostile.

Additionally concerning in our results was participants’ beliefs that international partners had prioritized diplomatic relations with the Tajik government over public health advocacy, suggesting misalignment in their priorities for HIV programming in the EECA region. While the role of international organizations has often been studied from the broader perspective of international development, study participants illuminated the specific and direct impact that these organizations had on HIV program delivery in Tajikistan. Existing research has critiqued international funding and programs for exacerbating “brain drain” to the private sector, imposing externally-driven priorities influenced by high-income country donors, and fragmenting and duplicating local efforts through new NGO creation [38, 39]. Research has also explored how civil society organizations adapt when these international organizations withdraw from a country [28]. However, limited attention has been given to the role of international organizations in mediating tensions between states and civil society. Our participants believed there had been a muted response from multilateral organizations and global health initiatives (e.g., UN agencies, Global Fund, PEPFAR) in reaction to the closure of NGOs in Tajikistan. In contrast, research from Africa [40] and Southeast Asia [41] has demonstrated the effectiveness of partnerships between international bodies and local NGOs in lobbying governments for reforms. Further investigation is needed to understand the reaction of international organizations in Tajikistan in this moment of change. This includes understanding how these organizations perceive their role amid increasingly restrictive funding landscapes and escalating state pressures.

Our findings provide actionable insights for designing and implementing future NGO-delivered HIV prevention interventions. As described earlier, there is limited evidence from the EECA region on external context within the PRISM framework as an implementation determinant, including on how government and international constraints influence delivery of HIV prevention programming. Here, we underscore the complex and critical role of external context in shaping civil society HIV programming and ability to conduct their work. When designing HIV prevention interventions to be delivered through NGOs, particularly in LMIC settings, program developers should prioritize understanding both national and international landscapes, and the complex relationships between various actors. This enables the design and delivery of more resilient and adaptable programs that can effectively navigate this external context. By ensuring flexibility and incorporating governance and funding considerations early in program development, interventions can be better positioned to overcome political barriers and maximize their reach and impact. Additionally, multilevel programs that strengthen NGO capabilities alongside specific HIV deliverables are essential and should be designed and tested specifically for the EECA context.

### Limitations

Our findings are subject to several limitations. First, due to the timing of our interviews in spring 2024, we did not collect participant reflections on the 2025 USAID and foreign aid cuts; global research suggests this will have drastic impacts on HIV prevention programming and lead to increased HIV incidence [42]. Continuous updates are needed to understand how these cuts are impacting local service delivery, as well as shifting planning and priorities within multilateral and international organizations. Social desirability bias may have encouraged participants to provide responses they perceived as more acceptable, potentially underreporting sensitive views or overemphasizing socially approved positions. In addition, the politically sensitive nature of the topic may have encouraged cautious or strategic responses from civil society sector participants, which could have affected both the candor of the data and the interpretation of some findings. Finally, despite extensive efforts, we were unable to recruit participants from UN agencies based in Dushanbe. This included non-response to interview requests for staff members at UNICEF, UNAIDS, and UNDP. The missing perspectives from these organizations are particularly acute, given the scope of their work and their partnerships with NGOs and the Tajik government. The absence of their perspectives limits the breadth of the analysis, as we may not have been aware of institutional viewpoints, operational constraints, and policy considerations that could have added important context to our findings.

## Conclusion

The challenges facing the NGO sector in Tajikistan pose a significant threat to the sustainability of existing HIV prevention efforts, with potentially far-reaching consequences for epidemic control. These findings underscore the need to support and leverage the unique strengths of Tajikistan's NGOs by addressing regulatory and funding challenges that currently constrain their ability to reach and deliver services to key populations.

## Supplementary Information

Supplementary Material 1.

## Data Availability

The study data contains potentially identifying information from study participants on a politically sensitive topic; therefore data are not openly available. Data can be made available from the corresponding author upon reasonable request.

## References

[1] UNAIDS. AIDSinfo | UNAIDS. 2023. https://aidsinfo.unaids.org/. Accessed 12 Dec 2023. Cited 12 Dec 2023.

[2] UNAIDS. Regional Profile: Eastern Europe and Central Asia. 2024. https://www.unaids.org/sites/default/files/media_asset/2024-unaids-global-aids-update-eeca_en.pdf. Accessed 11 Jul 2025. Cited 11 Jul 2025.

[3] Kaufman J. Civil Society Involvement in National HIV/AIDS Programs. HIVAIDS China. 2019;427–40. 10.1007/978-981-13-8518-6_22

[4] Kelly JA, Somlai AM, Benotsch EG, Amirkhanian YA, Fernandez MI, Stevenson LY, et al. Programmes, resources, and needs of HIV-prevention nongovernmental organizations (NGOs) in Africa, Central/Eastern Europe and Central Asia, Latin America and the Caribbean. AIDS Care. 2006;18:12–21. 10.1080/09540120500101757. 16282071 10.1080/09540120500101757 PMC2265204

[5] Mercer MA, Liskin L, Scott SJ. The role of non-governmental organizations in the global response to AIDS. AIDS Care. 1991;3:265–70. 10.1080/09540129108253072. 1932189 10.1080/09540129108253072

[6] Sikazwe I, Bolton-Moore C, Herce MB. Nongovernmental organizations supporting the HIV service delivery response in Africa - an engine for innovation. Curr Opin HIV AIDS. 2023;18:52–6. 10.1097/COH.0000000000000774. 36503879 10.1097/COH.0000000000000774

[7] Cornman H, Grimm CD, Rana S. Engaging Local Non-Governmental Organizations (NGOs) in the Response to HIV/AIDS.

[8] CADAP. Current situation of data collection and drug early warning system in Tajikistan. 2023. https://www.eu-cadap.org/wp-content/uploads/2024/04/Tajikistan-REPORT.pdf. Accessed 6 Apr 2025. Cited 6 Apr 2025.

[9] CABAR. Sex Work in Tajikistan: No one has the courage to legalize this activity. CABAR.asia. 2021. https://cabar.asia/en/sex-work-in-tajikistan-no-one-has-the-courage-to-legalize-this-activity. Accessed 6 Apr 2025. Cited 6 Apr 2025.

[10] Owczarzak J. Activism, NGOs, and HIV prevention in postsocialist Poland: the role of “anti-politics.” Hum Organ. 2010;69:200–11. 25308987 10.17730/humo.69.2.v8132n668713242k PMC4191668

[11] Kluczewska K. How to translate ‘good governance’ into Tajik? An American Good Governance Fund and norm localisation in Tajikistan. J Interv Statebuild. 2019;13:357–76. 10.1080/17502977.2018.1537668.

[12] Government of Tajikistan. Law of the Republic of Tajikistan on Public Associations (as amended by the Law of the Republic of Tajikistan dated March 20, 2008 No. 384, dated July 21, 2010 No. 621, dated March 19, 2013 No. 962, dated August 08, 2015 No. 1210, dated November 23, 2015 No. 1242, dated January 2, 2019. No. 1575). 2007.

[13] Hoare JP. Doing gender activism in a donor-organized framework: constraints and opportunities in Kyrgyzstan. Natl Pap. 2016;44:281–98. 10.1080/00905992.2015.1007344.

[14] International Center for Nonprofit Law. The Role of NGOs in Independent Tajikistan. ICNL. https://www.icnl.org/resources/research/ijnl/the-role-of-ngos-in-independent-tajikistan. Accessed 2 Mar 2025. Cited 2 Mar 2025.

[15] Kluczewska K. Questioning local ownership: insights from donor-funded NGOs in Tajikistan. J Civil Soc. 2019;15:353–72. 10.1080/17448689.2019.1668629.

[16] Spicer N, Harmer A, Aleshkina J, Bogdan D, Chkhatarashvili K, Murzalieva G, et al. Circus monkeys or change agents? Civil society advocacy for HIV/AIDS in adverse policy environments. Soc Sci Med. 2011;73:1748–55. 10.1016/j.socscimed.2011.08.024. 22036298 10.1016/j.socscimed.2011.08.024

[17] International Center for Nonprofit Law. Tajikistan - ICNL Civic Freedom Monitor. 2024 Nov. https://www.icnl.org/resources/civic-freedom-monitor/tajikistan. Accessed 17 Sept 2025

[18] Tysiachniouk M, Tulaeva S, Henry LA. Civil society under the law ‘on foreign agents’: NGO strategies and network transformation. Eur Asia Stud. 2018;70:615–37. 10.1080/09668136.2018.1463512.

[19] Gilbert L, Mohseni P. NGO laws after the colour revolutions and the Arab spring: nondemocratic regime strategies in Eastern Europe and the Middle East. Mediterr Polit. 2020;25:182–214. 10.1080/13629395.2018.1537103.

[20] Liu S. The Silenced Epidemic: Why Does Russia Fail to Address HIV? Georget. J. Int. Aff. 2022. https://gjia.georgetown.edu/2022/01/31/the-silenced-epidemic-why-does-russia-fail-to-address-hiv/. Accessed 2 Mar 2025. Cited 2 Mar 2025.

[21] AsiaPlus. B Taджикиcтaнe ликвидиpoвaли пoчти 700 HПO. Пoчeмy этo плoxo и кyдa идём? | Hoвocти Taджикиcтaнa ASIA-Plus. 2023. https://www.asiaplustj.info/ru/news/tajikistan/society/20230817/v-tadzhikistane-likvidirovalis-pochti-700-npo-pochemu-eto-ploho-i-kuda-idyom. Accessed 17 Sept 2025. Cited 17 Sept 2025.

[22] Feldstein AC, Glasgow RE. A practical, robust implementation and sustainability model (PRISM) for integrating research findings into practice. Jt Comm J Qual Patient Saf. 2008;34:228–43. 10.1016/s1553-7250(08)34030-6. 18468362 10.1016/s1553-7250(08)34030-6

[23] McHugh S, Dorsey CN, Mettert K, Purtle J, Bruns E, Lewis CC. Measures of outer setting constructs for implementation research: a systematic review and analysis of psychometric quality. Implement Res Pract. 2020;1:2633489520940022. 10.1177/2633489520940022. 37089125 10.1177/2633489520940022 PMC9924255

[24] Means AR, Kemp CG, Gwayi-Chore M-C, Gimbel S, Soi C, Sherr K, et al. Evaluating and optimizing the consolidated framework for implementation research (CFIR) for use in low- and middle-income countries: a systematic review. Implement Sci. 2020;15:17. 10.1186/s13012-020-0977-0. 32164692 10.1186/s13012-020-0977-0 PMC7069199

[25] Saunders B, Sim J, Kingstone T, Baker S, Waterfield J, Bartlam B, et al. Saturation in qualitative research: exploring its conceptualization and operationalization. Qual Quant. 2018;52:1893–907. 10.1007/s11135-017-0574-8. 29937585 10.1007/s11135-017-0574-8 PMC5993836

[26] LaDonna KA, Artino AR, Balmer DF. Beyond the guise of saturation: rigor and qualitative interview data. J Grad Med Educ. 2021;13:607–11. 10.4300/JGME-D-21-00752.1. 34721785 10.4300/JGME-D-21-00752.1 PMC8527935

[27] Braun V, Clarke V. Using thematic analysis in psychology. Qual Res Psychol. 2006;3:77–101. London, United Kingdom: Taylor & Francis Ltd. 10.1191/1478088706qp063oa.

[28] Pallas CL, Nguyen L. Donor withdrawal and local civil society organizations: An analysis of the HIV/AIDS sector in Vietnam. Dev Policy Rev. 2018;36:131–51. 10.1111/dpr.12236.

[29] Pallas CL, Sidel M. Foreign Aid Reduction and Local Civil Society: Recent Research and Policy Guidance for Donors and International NGOs. Nonprofit Policy Forum. De Gruyter; 2020;11. 10.1515/npf-2019-0045. Cited 8 Mar 2026.

[30] BTI 2024 Tajikistan Country Report. BTI 2024. https://bti-project.org/en/reports/country-report?isocode=TJK&cHash=500c49298883782af37b8ece58e99af1. Accessed 2 Mar 2025. Cited 2 Mar 2025.

[31] Tan RK, Wong CS. Mobilizing civil society for the HIV treatment cascade: a global analysis on democracy and its association with people living with HIV who know their status. J Int AIDS Soc. 2019;22:e25374. 10.1002/jia2.25374. 31379133 10.1002/jia2.25374 PMC6680091

[32] Gómez EJ, Harris J. Political repression, civil society and the politics of responding to AIDS in the BRICS nations. Health Policy Plan. 2016;31:56–66. 10.1093/heapol/czv021. 25858965 10.1093/heapol/czv021

[33] US Department of State. ForeignAssistance.gov. https://foreignassistance.gov/. Accessed 2 Apr 2025. Cited 2 Apr 2025.

[34] UNAIDS. Status of HIV programmes in Tajikistan | UNAIDS. https://www.unaids.org/en/resources/presscentre/featurestories/2025/march/20250305_Tajikistan_fs. Accessed 2 Apr 2025. Cited 2 Apr 2025.

[35] UNAIDS. Impact of US funding cuts on HIV programmes in Tajikistan | UNAIDS. https://www.unaids.org/en/resources/presscentre/featurestories/2025/march/20250319_Tajikistan_fs. Accessed 5 Apr 2025. Cited 5 Apr 2025.

[36] The impact of donor cuts on community-led responses in Tajikistan | UNAIDS. https://www.unaids.org/en/resources/presscentre/featurestories/2025/december/20251208_tajikistan. Accessed 3 Mar 2026. Cited 3 Mar 2026.

[37] Huffstetler HE, Bandara S, Bharali I, Kennedy Mcdade K, Mao W, Guo F, et al. The impacts of donor transitions on health systems in middle-income countries: a scoping review. Health Policy Plan. 2022;37:1188–202. 10.1093/heapol/czac063. 35904274 10.1093/heapol/czac063 PMC9558870

[38] Biesma RG, Brugha R, Harmer A, Walsh A, Spicer N, Walt G. The effects of global health initiatives on country health systems: a review of the evidence from HIV/AIDS control. Health Policy Plan. 2009;24:239–52. 10.1093/heapol/czp025. 19491291 10.1093/heapol/czp025 PMC2699244

[39] Ancker S, Rechel B. ‘Donors are not interested in reality’: the interplay between international donors and local NGOs in Kyrgyzstan’s HIV/AIDS sector. Diverging Paths Dev Cent Asia: Routledge; 2017.

[40] Nyoh IB. How multinational civil society organisations and non-governmental organisations lobby policy for human rights in Africa. J Public Aff. 2020;20:e1903. 10.1002/pa.1903.

[41] Pallas CL, Nguyen L. Transnational advocacy without northern NGO partners: Vietnamese NGOs in the HIV/AIDS sector. Nonprofit Volunt Sect Q. 2018;47:159S-176S. 10.1177/0899764018758462.

[42] Stone J, Mutai KK, Artenie A, Silhol R, Boily M-C, Ratevosian J, et al. The impact of cuts in the US President’s Emergency Plan for AIDS Relief funding for HIV pre-exposure prophylaxis in sub-Saharan Africa: a modelling study. Lancet HIV. 2025;12:e712–21. 10.1016/S2352-3018(25)00192-4. 41043881 10.1016/S2352-3018(25)00192-4 PMC7618903

