# Supplementary material for: How foreign and domestic pressures on NGOs shape service provision for HIV in Tajikistan: a qualitative analysis
Source: BMC Public Health. 2026 Apr 21;26:1301. doi: 10.1186/s12889-026-27450-w (PMC13097833; doi:10.1186/s12889-026-27450-w)
Supplement: Supplementary file 1 — Supplementary Material 1. [file 12889_2026_27450_MOESM1_ESM.docx]

**APPENDIX B**

**In-Depth Interview Guide for Key Informant Stakeholders**

**Script for Research Assistant (RA):**

*Thank you again for your participation in our study. We are interested in learning about HIV prevention, education, and testing services for youth and young adults, aged 15-24. In this interview, we’ll use the word “youth” to describe everyone in this age range. We want to better understand how you, and the organization you work for, provide programs or services, particularly those related to HIV. For example, we’ll be discussing what things make it easier or more challenging for you to provide such services, and what additional programming or resources you think are needed. Please remember that you don’t have to answer any questions that you are uncomfortable with, and you can choose to end this interview at any time.*

**[Turn on audio recorder]**

*Today is [date], it is [time], and I am [RA name]. This is Participant [Participant ID Number].*

*First, could I please ask you to confirm that you have already provided informed consent to take part in this interview?*

**If participant says “Yes”, continue to interview questions. Questions marked “Probe” are optional and can be skipped.**

1. *Can you start by describing your role at [your clinic/organization]?*
   1. *Probe: How long have you been working at [clinic/organization]?*
   2. *Probe: What roles or responsibilities have you held during that time?*
2. *For this interview, we’ll focus only on your AYA clients/patients, those aged 15-24. Can you tell me about the clients/patients that you work with in this age range?*
   1. *Probe: What gender are the majority of your youth clients?*
   2. *Probe: Education level?*
   3. *Probe: Economic status?*
   4. *Probe: Marital Status?*
3. *What are the first thoughts that come to mind when you think about HIV prevention among youth in Tajikistan?*
   1. *Probe: Are there any behaviors or characteristics that you associate with HIV risk/acquisition among youth?*
   2. *Probe: Statistically, sexual transmission is higher than injection drug use transmission – does this reflect what you see in the youth that you serve?*
   3. *Probe: Do you think youth ages 15-24 have access to condoms?*
   4. *Probe: Do you think that youth have access to HIV testing? What about with self-testing?*
4. *From your perspective, what are some of the challenges that your young patients or clients face when it comes to HIV education, prevention, and testing?*
   1. *Are your young clients/patients concerned about HIV infection?*
   2. *What are some different challenges for young men and young women?*
   3. *What are some different challenges between youth in urban areas like Dushanbe and youth in rural areas?*
5. *What is it like for you to work with youth?*
   1. *Can you describe how you interact differently with youth, compared to adults?*
   2. *Is there anything particularly challenging about working with youth?*
6. *In your opinion what makes a clinic/ organization welcoming and comfortable for youth?*
   1. *Probe: On the other hand, what makes a clinic unwelcoming or unfriendly?*
7. *When you work with youth aged 17 and younger, what is the role of the parents or guardians?*
   1. *What information about a child’s health (or about services received by the child) would you share with a child’s parents?*
   2. *What information about a child’s health (or about services received by the child) would you keep confidential and not share with the parents?*
   3. *What is the role of parents and guardians for youth who are 18 to 24?*
8. *I’d like to ask you briefly about stigma against HIV, and also stigma against associated risk behaviors, such as substance use and sex?*
   1. *Probe: How does community stigma (from family, friends, community) impact youth’s attitude towards HIV and their access to services?*
   2. *Probe: How does stigma from medical providers (doctors, nurses) in your organization or in other organizations impact their attitude towards HIV and their access to services.*

*We’ll now switch to discussing some of the HIV education and prevention work that you do at [name of clinic/organization]. We are interested in both regular services, and any special programs that you provide, or have provided recently. Again, please remember that we are particularly interested in programs that can be accessed by youth, age 15-24, and we are particularly interested in programs with a focus on HIV education or awareness raising, prevention activities like condom distribution, and HIV testing.*

1. *Can you tell me about some of the successes that you’ve had in providing HIV prevention services to youth?*
   1. *Probe: How does your organization define “success”? What data do you use to determine whether a service or program is “successful”?*
   2. *Probe: What policies, procedures, staffing, or leadership contributed to these successes?*
2. *Can you tell me some of the challenges that you’ve had in providing HIV prevention services to youth?*
   1. *Probe: What policies, procedures, staffing, or leadership contributed to these challenges?*
   2. *Probe: What strategies did you use to overcome these challenges?*
3. *In your opinion, are the current HIV prevention services that you offer at [clinic/organization] meeting the needs of youth in Tajikistan?*
   1. *If not, what new HIV programming or services would you like to be able to offer? Or what would you like to change?*
   2. *What services or programs do you think would be most effective for the prevention and/or detection of HIV among youth?*

*Next, I’ll ask you a few questions about your thoughts on new HIV prevention programs for youth and young adults.*

1. *What are your opinions on using internationally-developed (outside of Tajikistan) HIV prevention programs in your organization?*
   1. *Probe: What are the advantages and disadvantages of using internationally-developed programs?*
   2. *Probe: What adjustments would you need to make to these programs to make them appropriate for Tajikistan or appropriate for your organization?*
2. *What training can help doctors, nurses, social workers and others who do HIV prevention or testing work with youth? What content or skills should such trainings cover?*
3. *What data or information is helpful to clinics/organizations like yours when planning new services or programs for youth?*
4. *If you heard about a new training, program, or service that you think would be good for your clinic/organization, can you describe the steps you would take to realize it?*
   1. *Probe: Who would you need support from?*
   2. *Probe: Who would you need approval from?*
   3. *Probe: Apart from funding, would you need additional staff trainings, staff, space to implement a new program?*
5. *In your opinion, what makes a new service or program sustainable? By sustainable, we mean that it will be continued in the long term (5+ years)*

*I’d like to ask a few questions related to the working environment at your clinic/organization.*

1. *Can you describe the atmosphere at your clinic/organization?*
   1. *Probe: What are the management and communication structures in your clinic/organization?*
   2. *Probe: How are decisions made and communicated to all staff?*
   3. *Probe: How do staff provide feedback and ideas on new programs?*
   4. *Do employees of the organization take part in decision making?*
   5. What motivates the staff at your organization to do the work that you do?
2. Is there anything that we haven’t talked about today that you think is relevant or that we should know about HIV prevention and testing services among youth?

**Give participants time to ask questions before collecting sociodemographic data.**

*We’ll end with a few brief sociodemographic questions:*

*S1. What is your age:*

*S2. What is your gender?*

*S3. What is your ethnicity?*

(Provide options if needed: Tajik, Pamiri, Uzbek, Russian, Kyrgyz, Kazakh, mixed ethnicity etc.)

*S4. Can you please confirm the highest educational degree you have received?*

*S5. How many years have you provided HIV-related services?*

*Thank you very much for your participation in our interview.*

**[Stop recording and provide participant with compensation]**
